# Little Filtered Cigar, Cigarillo, and Premium Cigar Smoking Among Adults — United States, 2012–2013

**Published:** 2014-08-01

**Authors:** Catherine G. Corey, Brian A. King, Blair N. Coleman, Cristine D. Delnevo, Corinne G. Husten, Bridget K. Ambrose, Benjamin J. Apelberg

**Affiliations:** 1 Center for Tobacco Products, Food and Drug Administration; 2 Office on Smoking and Health, National Center for Chronic Disease Prevention and Health Promotion, CDC; 3 Center for Tobacco Studies, Rutgers School of Public Health

The burden of death and disease from tobacco use in the United States has been caused overwhelmingly by cigarettes and other smoked tobacco products (1). In the United States, cigarette consumption declined during 2000–2011; however, consumption of cigars more than doubled during the same period (2). The cigar market includes diverse product types manufactured with a variety of shapes and sizes, filters, tips, flavors, and prices (3). Although national estimates of cigar consumption have been reported previously (2,3), data characterizing who smokes different cigar types are limited. A recent analysis from the 2012–2013 National Adult Tobacco Survey (NATS) found that more than one in 20 U.S. adults smoke cigars “every day,” “someday,” or “rarely” (4). This report expands upon those findings, using data from the 2012–2013 NATS to further characterize cigar smokers by the usual type of cigar smoked using the following categories: little filtered cigars (LFCs), cigarillos/other mass market cigars (cigarillos/MMCs), and premium cigars. The findings indicate that among U.S. adults who smoke cigars, 61.8% usually smoke cigarillos/MMCs, 19.9% usually smoke premium cigars, and the remainder, 18.4%, usually smoke LFCs. These data can help to inform public health interventions to reduce the burden of adverse health effects caused by cigar smoking in the United States, including regulation.

The 2012–2013 NATS is a stratified, national, random-digit–dialed landline and cellular telephone survey of 60,192 noninstitutionalized U.S. civilian adults aged ≥18 years. The survey response rate was 44.9% (landline = 47.2%; cell phone = 36.3%). Respondents who now smoked cigars every day, some days, or rarely* were asked the following questions about the attributes of the cigar they usually smoked: 1) “Do you usually smoke a cigar, cigarillo, or little filtered cigar that has…A spongy filter/A plastic tip/A wooden tip/No filter or tip?”; and 2) “What is the name brand of the cigar, cigarillo, or little filtered cigar that you usually smoke?” LFC smokers were defined as those reporting their usual cigar had a spongy filter, or was from a manufacturer that primarily or exclusively manufactures LFCs. Premium cigar smokers were defined as those reporting their usual cigar did not have a filter or tip and the name of their usual brand was a brand name of a hand-rolled cigar (5) or a cigar described by the manufacturer or merchant as containing high-grade tobaccos in the filler, binder, or wrapper. Cigarillo/MMC smokers were defined as those reporting their usual cigar did not have a filter and the usual brand was not premium.†

Data were weighted to provide nationally representative estimates. Estimates of usual cigar type among current cigar smokers were calculated overall and by sex, age, race/ethnicity, U.S. Census region,§ education, annual household income, and sexual orientation. Estimates of cigar smoking frequency and cigarette smoking status (former, current, or never cigarette smoker) were calculated by usual cigar type.¶ Estimates with a relative standard error of ≥40% were omitted. Differences between groups were assessed using t-tests (p<0.05).

Among the 7.3% of U.S. adults who smoke cigars “every day,” “someday,” or “rarely,” more than half (52.5%) reported information that could be used to assign a usual cigar type. Of these cigar smokers, 61.8% usually smoke cigarillos/MMCs, 19.9% usually smoke premium cigars, and 18.4% usually smoke LFCs (Table). Cigarillos/MMCs were the usual cigar of most men and most women. Premium cigars were the usual cigar of 23.9% of men, and LFCs were the choice of more women (35.3%) than men (14.5%) (p<0.05). Cigarillos/MMCs were the usual cigar of 72.1% of young adults (aged 18–29 years) but were less popular among older adults. However, an estimated 15.1% of persons aged 18–29 years smoked premium cigars, which was comparable to LFCs (12.8%). By race/ethnicity, cigarillos/MMCs were the usual type among cigar smokers who were non-Hispanic black (82.6%), among whom premium cigar smoking was the usual type for only 5.2%. In contrast, among non-Hispanic whites, 26.7% reported premium cigars as their usual choice. In the Northeast, unlike other regions, premium cigars were the usual cigar of more cigar smokers than were LFCs. Higher educational levels and annual household income generally were associated with lower prevalence of usual use of cigarillos/MMCs and of LFCs, and higher prevalence of usual use of premium cigars. By sexual orientation, prevalence of LFCs as a usual cigar was greater among lesbian, gay, and bisexual adults (35.6%) than among heterosexual/straight adults (17.6%) (p<0.05).

Among cigar smokers who usually smoked premium cigars, 3.3% reported “every day” use, 25.6% reported “some day” use, and 71.2% reported use “rarely” (Figure 1). Among usual smokers of cigarillos/MMCs, 13.3% reported “every day” use, 23.0% reported “some day” use, and 63.8% reported use “rarely.” Among usual smokers of LFCs, 36.0% reported “every day” use, 21.5% reported “some day” use, and 42.5% reported use “rarely.”

Among usual smokers of premium cigars, 35.1% currently smoked cigarettes, 23.0% formerly smoked cigarettes, and 41.9% never smoked cigarettes (Figure 2). Among usual cigarillo/MMC smokers, 58.3% currently smoked cigarettes, 15.3% formerly smoked cigarettes, and 26.4% never smoked cigarettes. Among usual LFC smokers, 75.2% smoked cigarettes, 12.3% formerly smoked cigarettes, and 12.4% never smoked cigarettes.

## Discussion

On the basis of these results, it is estimated that three in five cigar smokers (an estimated 10.9 million persons) usually smoke cigarillos/MMCs, approximately one in five (an estimated 3.6 million persons) usually smoke premium cigars, and nearly one in five (an estimated 2.9 million persons) usually smoke LFCs. Younger adults were more likely to identify cigarillos/MMCs as their usual cigar (72.1% of cigar smokers). However, among those aged 18–29 years, 15.1% reported that their usual cigar was a premium cigar; 12.7% usually smoked LFCs. Additionally, variations in usual cigar type by sex, age, race/ethnicity, education, income, and sexual orientation suggest that a single, aggregated measure of cigar smoking might mask important differences in tobacco use behaviors. For example, more than eight in 10 cigar smokers who were non-Hispanic black used cigarillos/MMCs. More disaggregated surveillance by cigar type has the potential to better inform public health strategies to reduce the health burden of cigar smoking in the United States. Regular cigar use is estimated to be responsible for approximately 9,000 premature deaths and almost 140,000 years of potential life lost annually, and this might underestimate total premature mortality because deaths resulting from less frequent cigar smoking were not estimated (6). These findings underscore the importance of public health interventions to reduce cigar smoking among U.S. adults. Evidence-based tobacco control interventions such as increased taxes, smoke-free policies, and public education campaigns should also address noncigarette tobacco products.

What is already known on this topic?

The cigar market includes diverse product types manufactured with a variety of shapes and sizes, filters, tips, flavors, and prices. Although national estimates of cigar consumption have been reported previously, data characterizing who uses different cigar types are limited.

What is added by this report?

Among current cigar smokers who provided information that could be used to assign a usual cigar type, 61.8% usually smoke cigarillos/other mass market cigars, 19.9% usually smoke premium cigars, and the remainder, 18.4%, usually smoke little filtered cigars. Variations in usual cigar type were observed by sex, age, race/ethnicity, education, household income, and sexual orientation. Daily cigar smoking was reported by 3.3% of usual premium cigar smokers, 13.3% of usual cigarillo/other mass market cigar smokers, and 36.0% of usual little filtered cigar smokers. Among each group of smokers, nearly 60% or more of current cigar smokers are either current or former cigarette smokers; these persons are more likely to inhale cigar smoke more deeply, putting them at particularly high risk for tobacco-related diseases.

What are the implications for public health practice?

Enhanced monitoring of noncigarette product use, including use of different cigar types and concurrent use of multiple tobacco products, can inform efforts to reduce the burden of adverse health effects caused by cigar smoking in the United States.

Daily cigar smoking was reported by 3.3% of usual premium cigar smokers, 13.3% of usual cigarillo/MMC smokers, and 36.0% of usual LFC smokers, suggesting that cigar smoking is not exclusively an infrequent or rare behavior. Regardless of cigar type, nearly 60% or more of current cigar smokers are either current or former cigarette smokers. Cigar smokers that are current or former cigarette smokers are more likely to report inhaling cigar smoke, putting them at particularly high risk for tobacco-related diseases (7). Even cigar smokers who report not inhaling show evidence of inhalation (8). Moreover, elevated risks for dying from oral, pharyngeal, laryngeal, and esophageal cancers have been found among those reporting no inhalation (7). The high proportion of current and former cigarette smoking reported by current cigar smokers underscores the importance of continued implementation of proven, population-based interventions to address all forms of tobacco use, especially smoked products such as cigarettes and cigars that currently account for the greatest public health burden in the United States (1).

The findings in this report are subject to at least six limitations. First, cigar use was self-reported and not biochemically verified; however, self-reported cigarette smoking correlates highly with serum cotinine levels, suggesting good validity of self-reported cigar use (9). Second, more than 47% of current cigar smokers could not be assigned a usual cigar type because of insufficient information about the usual cigar smoked; to generate weighted counts of cigar smokers by cigar type, the distribution of usual type was assumed to be the same for those with and without missing information, after controlling for frequency of use. Third, for each type of cigar, data were gathered only from persons who smoked them as their usual cigar; persons who smoked more than one type of cigar contributed data only for their usual cigar type. Fourth, price or cigar weight could also be used to differentiate cigar types; however, NATS did not collect data for either. Fifth, small sample sizes for certain subgroups resulted in less precise estimates. Finally, the NATS response rate of 44.9% might have resulted in nonresponse bias, even after adjustment for nonresponse. Although they are not limitations, it is important to note two features of the 2012–2013 NATS. First, a response option of “rarely” was provided to characterize cigar smoking frequency after cognitive testing suggested some cigar smokers did not consider “every day,” “some days,” or “not at all” to accurately reflect their use; however, although respondents chose this option to describe their current use of cigars, exactly how respondents interpreted “rarely” is unknown. In addition, the approach used to assign usual cigar type differed from that of another national tobacco survey, which asked cigar smokers to report their usual cigar type as either LFC, cigarillo, or regular/large cigar, thus yielding potentially different distributions of cigar smokers according to usual cigar type (10).

In April 2014, the Food and Drug Administration proposed to extend its jurisdiction over the manufacture, marketing, and sale of tobacco products to cigars.** Full implementation of comprehensive tobacco control programs at CDC-recommended funding levels could reduce all forms of tobacco use, including cigars, and change social norms regarding the acceptability of tobacco use in the United States.†† Additionally, given the diversity of tobacco product use in the United States (4), enhanced surveillance of noncigarette products, including different cigar types and concurrent use of multiple tobacco products, is critical.

## Figures and Tables

**FIGURE 1:**
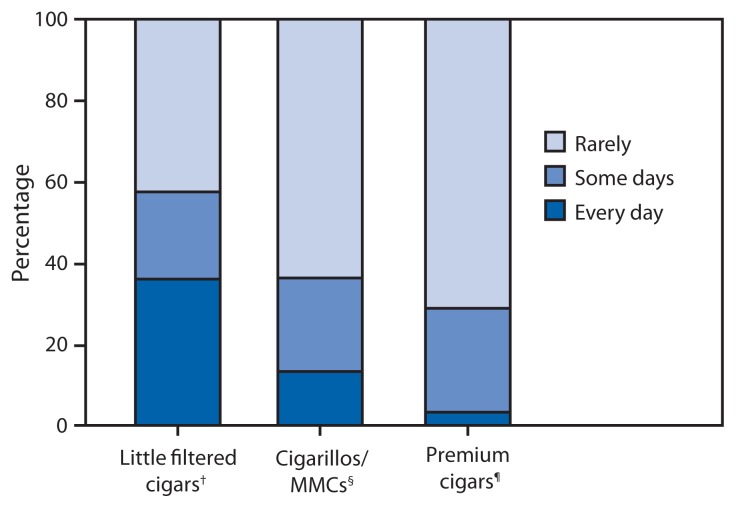
Percentage of current cigar smokers aged ≥18 years who smoke cigars every day, some days, or rarely, by type of cigar usually smoked — National Adult Tobacco Survey, United States, 2012–2013 **Abbreviation:** MMC = mass market cigar. * To be eligible to be assigned a usual cigar type, respondents had to currently smoke cigars “every day,” “some days,” or “rarely”; in addition, adults aged ≥30 years had to report smoking ≥50 cigars in their lifetime, whereas adults aged 18–29 years did not. ^†^ Respondent reported their usual cigar had a spongy filter, or was from a manufacturer that primarily or exclusively manufactures little filtered cigars. ^§^ Respondent reported their usual cigar did not have a filter and the usual brand was not premium. ^¶^ Respondent reported their usual cigar did not have a filter or tip and the name of their usual brand was a brand name of a hand-rolled cigar or a cigar described by the manufacturer or merchant as containing high-grade tobaccos in the filler, binder, or wrapper.

**FIGURE 2:**
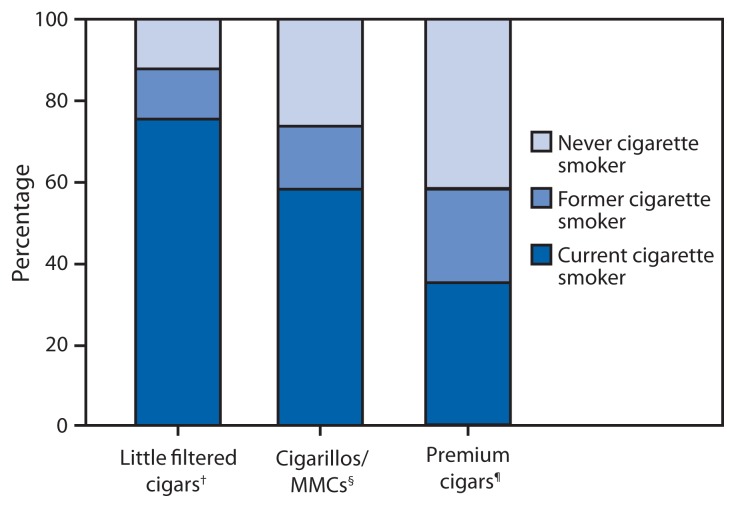
Percentage of current cigar smokers aged ≥18 years who currently smoke cigarettes, formerly smoked cigarettes, or never smoked cigarettes, by type of cigar usually smoked — National Adult Tobacco Survey, United States, 2012–2013 **Abbreviation:** MMC = mass market cigar. *To be eligible to be assigned a usual cigar type, respondents had to currently smoke cigars “every day,” “some days,” or “rarely”; in addition, adults aged ≥30 years had to report smoking ≥50 cigars in their lifetime, whereas adults aged 18–29 years did not. ^†^Respondent reported their usual cigar had a spongy filter, or was from a manufacturer that primarily or exclusively manufactures little filtered cigars. ^§^Respondent reported their usual cigar did not have a filter and the usual brand was not premium. ^¶^Respondent reported their usual cigar did not have a filter or tip and the name of their usual brand was a brand name of a hand-rolled cigar or a cigar described by the manufacturer or merchant as containing high-grade tobaccos in the filler, binder, or wrapper.

**TABLE:** Selected characteristics of current cigar smokers* aged ≥18 years who identified a usual type of cigar smoked, by usual cigar type — National Adult Tobacco Survey, United States, 2012–2013

	Little filtered cigars†		Cigarillos/MMCs§		Premium cigars¶	
			
Characteristic	%	(95% CI)	%	(95% CI)	%	(95% CI)
**Overall**	**18.4**	**(16.1–20.9)**	**61.8**	**(58.6–64.9)**	**19.9**	**(17.4–22.6)**
**Sex**						
Men	14.5	(12.1–17.2)	61.6	(57.9–65.2)	23.9	(21.0–27.2)
Women	35.3	(28.7–42.4)	59.4	(52.0–66.5)	—††	
**Age group (yrs)**						
18–29	12.8	(9.8–16.5)	72.1	(67.2–76.5)	15.1	(11.7–19.2)
30–49	18.5	(14.5–23.4)	57.6	(51.6–63.3)	23.9	(19.1–29.5)
50–64	31.7	(26.2–37.8)	43.5	(37.7–49.4)	24.8	(20.4–29.8)
≥65	24.0	(17.5–32.1)	55.6	(46.8–64.1)	20.4	(14.6–27.7)
**Race/Ethnicity**						
White, non-Hispanic	20.2	(17.2–23.6)	53.1	(48.9–57.2)	26.7	(23.2–30.5)
Black, non-Hispanic	12.2	(7.5–19.3)	82.6	(74.8–88.3)	5.2	(2.5–10.4)
Other, non-Hispanic	22.5	(16.3–30.2)	68.4	(60.2–75.6)	9.1	(5.8–14.1)
Hispanic	14.7	(8.4–24.4)	65.9	(54.0–76.0)	19.5	(11.5–30.9)
**U.S Census region** **						
Northeast	13.9	(9.7–19.7)	57.6	(48.2–66.4)	28.5	(20.4–38.3)
Midwest	20.2	(15.5–25.9)	65.2	(58.7–71.2)	14.6	(10.8–19.6)
South	16.3	(13.1–20.2)	64.8	(59.9–69.3)	18.9	(15.5–22.9)
West	23.1	(17.3–30.1)	57.0	(49.7–64.0)	19.9	(15.4–25.2)
**Education**						
0–12 years (no diploma)	23.6	(16.8–32.0)	73.1	(64.2–80.4)	—††	
High school diploma or GED	19.0	(14.9–24.0)	69.8	(63.7–75.3)	11.1	(7.3–16.6)
Some college or associate degree	20.5	(16.5–25.0)	57.1	(51.6–62.4)	22.5	(18.1–27.5)
Undergraduate degree or higher	8.0	(5.3–11.8)	40.3	(34.6–46.3)	51.7	(45.8–57.6)
**Annual household income ($)**						
<20,000	34.6	(27.5–42.5)	60.6	(52.5–68.1)	4.8	(2.5–8.9)
20,000–49,999	17.6	(13.7–22.2)	71.5	(66.0–76.5)	10.9	(7.8–15.1)
50,000–99,999	12.1	(8.5–17.1)	60.9	(53.8–67.5)	27.0	(21.3–33.6)
≥100,000	10.1	(6.0–16.6)	49.9	(41.8–58.0)	40.0	(32.4–48.1)
Unspecified	21.0	(15.5–28.0)	58.3	(50.4–65.8)	20.7	(14.8–28.2)
**Sexual orientation**						
Heterosexual/Straight	17.6	(15.2–20.2)	61.5	(58.0–64.9)	20.9	(18.2–23.9)
Lesbian, gay, bisexual	35.6	(22.5–51.2)	51.9	(36.8–66.6)	12.5	(5.8–25.0)
Unspecified	18.1	(11.6–27.2)	67.0	(56.6–76.0)	14.9	(8.5–24.8)

**Abbreviations:** CI = confidence interval; MMC = mass market cigar; GED = General Education Development certification.

* To be eligible to be assigned a usual cigar type, respondents had to now smoke cigars “every day,” “some days,” or “rarely”; in addition, adults aged ≥30 years had to report smoking ≥50 cigars in their lifetime, whereas adults aged 18–29 years did not.

† Respondent reported their usual cigar had a spongy filter, or was from a manufacturer that primarily or exclusively manufactures little filtered cigars. Respondents who smoked little filtered cigars but usually smoked another type are not included.

§ Respondent reported their usual cigar did not have a filter and the usual brand was not premium. Respondents who smoked cigarillos/other mass market cigars but usually smoked another type are not included.

¶ Respondent reported their usual cigar did not have a filter or tip, and the name of the usual brand was identified as being hand-rolled or otherwise described as containing high-grade tobaccos in the filler, binder, or wrapper. Respondents who smoked premium cigars but usually smoked another type are not included.

** *Northeast*: Connecticut, Maine, Massachusetts, New Hampshire, New Jersey, New York, Pennsylvania, Rhode Island, and Vermont. *Midwest*: Illinois, Indiana, Iowa, Kansas, Michigan, Minnesota, Missouri, Nebraska, North Dakota, Ohio, South Dakota, and Wisconsin. *South*: Alabama, Arkansas, Delaware, District of Columbia, Florida, Georgia, Kentucky, Louisiana, Maryland, Mississippi, North Carolina, Oklahoma, South Carolina, Tennessee, Texas, Virginia, and West Virginia. *West*: Alaska, Arizona, California, Colorado, Hawaii, Idaho, Montana, Nevada, New Mexico, Oregon, Utah, Washington, and Wyoming.

†† Estimate not presented because relative standard error ≥40%.

## Footnotes

* Respondents aged ≥30 years had to have reported smoking ≥50 cigars in their lifetime to be asked the questions about cigar attributes, whereas respondents aged 18–29 years did not.

† The 2012–2013 NATS asked a third question about the size of the usual cigar smoked. Whereas respondents who reported using a little filtered cigar or a premium brand tended to report a consistent length for their usual cigar, cigarillo/MMC smokers did not; hence, length information was not used to assign usual cigar type. Among current cigar smokers, 48% could not be assigned a usual cigar type because of insufficient information. Two sources accounted for more than 90% of the missing data. Two thirds not assigned a usual cigar type responded “I do not have a usual size of cigar” to a question about length of the usual cigar smoked and were not asked the cigar filter/tip or brand questions; more than 80% of these users reported smoking cigars rarely. Another 27% reported smoking a cigar that did not have a filter or tip, but did not provide a usual cigar brand, and consequently could not be distinguished as a cigarillo/MMC or premium cigar smoker.

§ *Northeast:* Connecticut, Maine, Massachusetts, New Hampshire, New Jersey, New York, Pennsylvania, Rhode Island, and Vermont. *Midwest:* Illinois, Indiana, Iowa, Kansas, Michigan, Minnesota, Missouri, Nebraska, North Dakota, Ohio, South Dakota, and Wisconsin. *South:* Alabama, Arkansas, Delaware, District of Columbia, Florida, Georgia, Kentucky, Louisiana, Maryland, Mississippi, North Carolina, Oklahoma, South Carolina, Tennessee, Texas, Virginia, and West Virginia. *West:* Alaska, Arizona, California, Colorado, Hawaii, Idaho, Montana, Nevada, New Mexico, Oregon, Utah, Washington, and Wyoming.

¶ Respondents who reported smoking ≥100 cigarettes in their lifetime and reported now smoking every day or some days were recoded as current cigarette smokers. Respondents aged 18–29 years who reported not smoking ≥100 cigarettes in their lifetime but reported now smoking cigarettes every day or some days were recoded as current smokers. Respondents who reported smoking ≥100 cigarettes in their lifetime and reported now smoking not at all were recoded as former cigarette smokers. Respondents who reported not currently smoking at all and reported not smoking ≥100 cigarettes in their lifetime were recoded as never cigarette smokers.

** Additional information available at http://www.fda.gov/aboutfda/reportsmanualsforms/reports/economicanalyses/ucm394922.htm.

†† Additional information available at http://www.cdc.gov/tobacco/stateandcommunity/best_practices.
